# Sortilin levels are associated with peripheral arterial disease in type 2 diabetic subjects

**DOI:** 10.1186/s12933-019-0805-5

**Published:** 2019-01-11

**Authors:** Federico Biscetti, Nicola Bonadia, Francesco Santini, Flavia Angelini, Elisabetta Nardella, Dario Pitocco, Angelo Santoliquido, Marco Filipponi, Raffaele Landolfi, Andrea Flex

**Affiliations:** 1 U.O.C. Clinica Medica e Malattie Vascolari, Department of Medicine, Fondazione Policlinico Universitario A. Gemelli IRCCS, Largo F. Vito 1, 00168 Rome, Italy; 2 Laboratory of Vascular Biology and Genetics, Università Cattolica del Sacro Cuore, Rome, Italy; 3 U.O.C. Medicina d’Urgenza e Pronto Soccorso, Fondazione Policlinico Universitario A. Gemelli IRCCS, Rome, Italy; 4 U.O.S.A. di Diabetologia, Fondazione Policlinico Universitario A. Gemelli IRCCS, Rome, Italy; 5 Università Cattolica del Sacro Cuore, Rome, Italy; 6 U.O.S. Angiologia Columbus, Fondazione Policlinico Universitario A. Gemelli IRCCS, Rome, Italy; 7 Ospedale San Giovanni Battista ACISMOM, Rome, Italy

**Keywords:** Sortilin, Type 2 diabetes mellitus, Lower limb peripheral artery disease

## Abstract

**Background:**

Sortilin is a 95-kDa protein which has recently been linked to circulating cholesterol concentration and lifetime risk of developing significant atherosclerotic disease. Sortilin is found inside different cell types and circulating in blood. Higher circulating sortilin concentration has been found in patients with coronary atherosclerosis compared to control subjects. Sortilin concentration is influenced by statin therapy.

**Methods:**

We enrolled statin-naïve subjects with type 2 diabetes mellitus and we performed a cross-sectional study to evaluate the association between sortilin levels and the presence of clinically significant lower limb peripheral artery disease (PAD) in a population of statin-free diabetic subjects.

**Results:**

Out of the 154 patients enrolled in our study, 80 patients were free from PAD, while 74 had clinically significant PAD. Sortilin concentration was significantly higher in the latter group compared to the former (1.61 ± 0.54 ng/mL versus 0.67 ± 0.30 ng/mL, P < 0.01) and there was a trend toward increased sortilin levels as disease severity increased. The association of sortilin levels with PAD remained after adjusting for major risk factors in a multivariate analysis.

**Conclusions:**

We showed that sortilin is significantly and independently associated with the presence of lower limb PAD in a statin-free diabetic population and it may be a promising marker for clinically significant atherosclerosis of the lower limbs. Further studies are needed to confirm this finding and to evaluate its clinical usefulness.

## Background

Sortilin, a 95-kDa protein mainly expressed in hepatocytes, takes part in intracellular protein sorting between the trans-Golgi network and endosomes [1]. In a lesser quantity, it is also expressed on cell membrane and small quantities of sortilin have been found in circulating form. Sortilin is codified on chromosome 13 by the gene SORT1, whose polymorphisms have been linked with circulating levels of low-density lipoprotein (LDL) cholesterol and with the lifetime risk of developing clinically significant atherosclerosis [2–12].

Sortilin seems to take part in apolipoproteins trafficking inside hepatocytes, binding to apoB100 and promoting its trafficking to endosomal system, thereby promoting its degradation and reducing circulating levels of very low-density lipoprotein (VLDL). Moreover, membrane sortilin acts as a receptor for circulating LDL, promoting their uptake by hepatocytes via an LDL-receptor (R)-independent mechanism [13–17]. After the discovery of its link to atherosclerosis, there has been considerable interest in elucidating additional functions of sortilin in different cell types. Thus, sortilin has been discovered to play a role in intracellular cytokine traffic [18, 19] and to be expressed in platelets, from where it is released after activation [20]. Among those additional roles, the sortilin function as a membrane LDL-R has been implicated in LDL uptake by macrophages and in foam cell formation and directly in inflammatory mechanisms during atherosclerotic plaque formation and progression [21, 22]. Additionally, sortilin seems to be a fundamental player in hepatic and muscular response to insulin and may be a missing link between insulin resistance and hypercholesterolemia [23–25].

Aside from its potential as a therapeutic target, sortilin has been found in increased quantities in patients with coronary artery disease (CAD) and in patients with CAD risk factors compared to control subjects [20, 26].

Peripheral artery disease (PAD) is a well-known manifestation of atherosclerosis, which is associated with considerable disability and mortality. Lower limb PAD (LL-PAD) is of particular concern in diabetic patients and in active or current smokers, where the incidence and prevalence of the disease are particularly high. Ankle-brachial index (ABI) is currently recommended as the main screening tool for PAD in diabetic patients and in those with multiple risk factors [27]. A reduction in ABI, however, represents an already clinically significant stage of the disease. A tool used to find patients with higher risk of developing significant LL-PAD would be useful in identifying patients at a particularly high risk of developing clinically significant PAD and in tailoring more aggressive preventive and diagnostic strategies.

Considering the already proven links between the sortilin and multiple aspects of atherosclerosis [28, 29], metabolic imbalance [30] and diabetes [31], it is possible to hypothesize a connection between sortilin and PAD in the diabetic scenario.

## Methods

The aim of this study was to assess the possible role of sortilin as an easy-to-measure marker for the presence of LL-PAD in an in-patient population with type 2 diabetes mellitus (T2DM).

### Study design

We performed a cross-sectional study which was approved by the Ethics Committee of the *Fondazione Policlinico Universitario A. Gemelli IRCCS* and adhered to the principles of the Declaration of Helsinki. All the individuals agreed to participate in the study and gave informed consent. We analyzed the diabetic patients consecutively admitted to the Department of Vascular Diseases of the *Fondazione Policlinico Universitario A. Gemelli IRCCS*, Roma, Italy, from October 1, 2015 to January 30, 2018. Each patient admitted to the unit during the study time was evaluated for the enrollment in the present study. To be enrolled, each patient had to fulfill the criteria shown in Table 1.

**Table 1:** Inclusion and exclusion criteria

Inclusion criteria	Exclusion criteria
18 years and older	Undefined type of diabetes or clinical suspicion of non-type 2 diabetes mellitus
Confirmed diagnosis of type 2 diabetes mellitus at the time of admission	Confirmed pancreatic insufficiency, chronic pancreatitis or previous pancreatic surgery
Able to understand study procedures	Ongoing steroidal therapy or steroidal therapy in the previous 3 months
Willing to participate in the study and to sign the written informed consent	Ongoing calcineurin inhibitor therapy or history of calcineurin inhibitor therapy
	Ongoing statin therapy or statin therapy in the previous month
	Suspected or confirmed pregnancy
	Chronic kidney disease with eGFR below 30 mL/min (according to the CKD-EPI equation)
	Previous non-traumatic lower limb amputation
	Solid organ or marrow transplant recipient
	History or active solid or hematological malignancy
	Acute infectious disease at the time of evaluation or in the previous month
	Chronic liver disease with a functional status, according to the Child–Pugh classification, of B or above
	Hereditary monogenic dyslipidemia (confirmed or suspected)
	Congenital disease of platelet function
	Acquired or congenital thrombocytopenia
	Congenital or acquired hemophilia or coagulation defect
	Congenital or acquired thrombophilia
	Active autoimmune disease

We enrolled diabetic patients with or without PAD. Type 2 diabetes mellitus was defined as a fasting plasma glucose ≥ 126 mg/dL and/or HbA1c ≥ 6.5% or as medical history for the presence of diabetes plus treatment with diabetes medication. Each patient enrolled in the study had his/her medical history assessed for the presence of PAD symptoms or a confirmed PAD diagnosis. For each diabetic patient evaluated in the study, ABI was performed. Patients with clinical findings consistent with PAD underwent ABI measurement and either lower limb arterial Doppler-enhanced ultrasonography, lower limb angiography or computed tomography (CT)-angiography, at the attending physician’s judgment. The patients with a > 0.90 ABI measurement and without symptoms of PAD did not undergo further testing and were deemed to be without PAD.

The patients were considered to have PAD if:

- They had had a history of previous lower limb percutaneous transluminal angioplasty, with or without stent placement, or
- They had had at least one instrumental and one clinical criterion among those listed in Table 2.

**Table 2:** Criteria for PAD definition in patients without a history of lower limb amputation, PTA or by-pass surgery

Clinical Criteria	Instrumental criteria
Presence of *claudication intermittens*	ABI < 0.90
Rest pain	TcPO_2_ < 30 mmHg
Non-healing distal ulcer	Ultrasonographic or radiologic finding of atherosclerotic narrowing, with a reduction of at least 50% of the lumen diameter, consistent with clinical symptoms
Gangrene	Ultrasonographic finding of post-stenotic blood flow profile, consistent with symptoms

Such criteria were consistent with published literature [32–35].

The extent of PAD was determined by using the Fontaine classification, which defines four stages:

Stage I, asymptomatic; stage II, intermittent claudication; stage III, rest pain; stage IV ischemic ulcers or gangrene [36, 37].

### Biochemical measurement

All patients enrolled underwent a blood test after an overnight fasting period of 8 h. For every patient, fasting glucose, triglycerides, total cholesterol, low and high-density lipoprotein, creatinine, aspartate aminotransferase, alanine aminotransferase, total bilirubin, alkaline phosphatase and complete blood count were determined. Renal function was assessed using estimated glomerular filtration rate (eGFR), which was calculated using the modification of diet in renal disease (MDRD) formula, as previously described [38]. Serum obtained and separated by centrifugation of blood samples was stored at − 80 °C before every measurement. Serum sortilin level were determined by a commercially available ELISA kit (RAB1709 SIGMA, Sigma-Aldrich) according to its protocol. The intra and inter-assay coefficients of variation were 3.5 and 10.5%, respectively. The sensitivity, defined as the mean ± 3 SD of the 0 standard, was calculated to be 0.15 pmol/mL. For each patient, the serum levels were measured twice and the results were averaged.

### Statistical analysis

Demographic and clinical data between the groups were compared using Chi squared and t tests. Sortilin serum levels were compared through Mann–Whitney test. A log transformation was applied to not-normally distributed variables (fasting glucose, glycated hemoglobin, triglyceride, and sortilin levels) prior to performing further analysis. A multivariate stepwise logistic regression analysis was performed, adjusted for traditional risk factors and sortilin levels. The area under the receiver-operating characteristics (ROC) curve was calculated to test its predictive discrimination of PAD. All analyses were performed using the STATA version 11.0 for Windows (Statistics/Data Analysis, Stata Corporation, College Station, TX, USA). Statistical significance was established at P < 0.05.

## Results

During the study period, 154 patients with a confirmed diagnosis of T2DM and not on statin therapy in the last 30 days, were included in the study. Eighty of the patients did not fulfill the criteria for a diagnosis of PAD, while the remaining 74 had a confirmed diagnosis of PAD or received a diagnosis of PAD during the index hospitalization. The demographic and clinical characteristics of the two groups are reported in Table 3. PAD patients had higher blood pressure values (P = 0.024), were more often smokers (P = 0.032) and were affected by CAD (defined as history of ischemic heart disease and/or previous coronary revascularization) more frequently (P = 0.031). There were no significant differences between groups regarding body mass index (BMI) (P = 0.52), median duration of diabetes (P = 0.97), fasting glucose (P = 0.75), glycated hemoglobin (P = 0.59), total cholesterol (TC) (P = 0.78), HDL-cholesterol (P = 0.67), LDL-cholesterol (P = 0.73) and triglyceride (P = 0.99). No statistical difference in terms of eGFR (P = 0.12), aspartate aminotransferase (P = 0.61), alanine aminotransferase (P = 0.45), total bilirubin (P = 0.33) and alkaline phosphatase (P = 0.56). Furthermore, there were no significant differences between groups regarding the complete blood count (Table 3). No statistical difference in terms of diabetic therapy was observed between the two patient groups. According to the Fontaine’s classification 36 patients were defined as stage II, 24 as stage III and 14 as stage IV.

**Table 3:** Demographic and clinical data of diabetic subjects with and without PAD

	WPAD	PAD	P value
	(n = 80)	(n = 74)	
Male (%)	69	65	0.34
Age (years ± SD)	73.1 ± 6.4	69.8 ± 6.3	0.89
Body mass index (kg/m^2^)	24.3 ± 2.1	23.9 ± 2.8	0.52
Smoking (current) (%)	17 (21.25)	19 (25.67)	0.032
Hypertension (%)	35 (44.0)	47 (63.5)	0.024
Coronary artery disease (%)	25 (31.2)	39 (52.7)	0.031
Diabetes duration (years ± SD)	10.1 ± 6.1	11.6 ± 5.9	0.97
Total cholesterol (mmol/L)	5.97 (1.81)	5.87 (1.35)	0.78
HDL-cholesterol (mmol/L)	1.24 (1.21)	1.20 (1.21)	0.67
LDL-cholesterol (mmol/L)	2.82 (1.08)	2.78 (0.83)	0.73
Triglyceride (mmol/L)	2.32 (1.67)	1.98 (1.12)	0.99
Fasting glucose (mmol/L)	7.19 (1.12)	7.61 (1.85)	0.75
Glycated hemoglobin (%)	7.13 (1.41)	7.21 (1.84)	0.59
eGFR (mL/min per 1.73 m^2^)	72.12 (8.19)	67.98 (6.76	0.12
Aspartate aminotransferase (UI/L)	38.2 (4.5)	34.4 (3.8)	0.61
Alanine aminotransferase (UI/L)	41.1 (4.9)	44.3 (3.9)	0.45
Total bilirubin (mg/dL)	0.9 (0.3)	1.1 (0.4)	0.33
Alkaline phosphatase (UI/L)	109.6 (12.3)	112.3 (9.4)	0.56
Hemoglobin (g/dL)	11.6 (2.12)	12.1 (2.01)	0.98
White blood cells (× 10E3/μL)	5.9 (0.68)	6.1 (0.98)	0.85
Platelets (× 10E3/μL)	254 (15.2)	236 (17.3)	0.52
Treatment			
Diet only (%)	9 (11.25)	11 (14.86)	0.76
Oral agents (%)	42 (52.5)	38 (51.35)	0.88
Insulin therapy (%)	38 (47.5)	35 (47.29)	0.65
PAD			
1-Fontaine’s II (%)		36 (48.64)	
2-Fontaine’s III (%)		24 (32.43)	
2-Fontaine’s IV (%)		14 (18.91)	

Data are number (%) and standard deviation (SD)

**Fig. 1:**
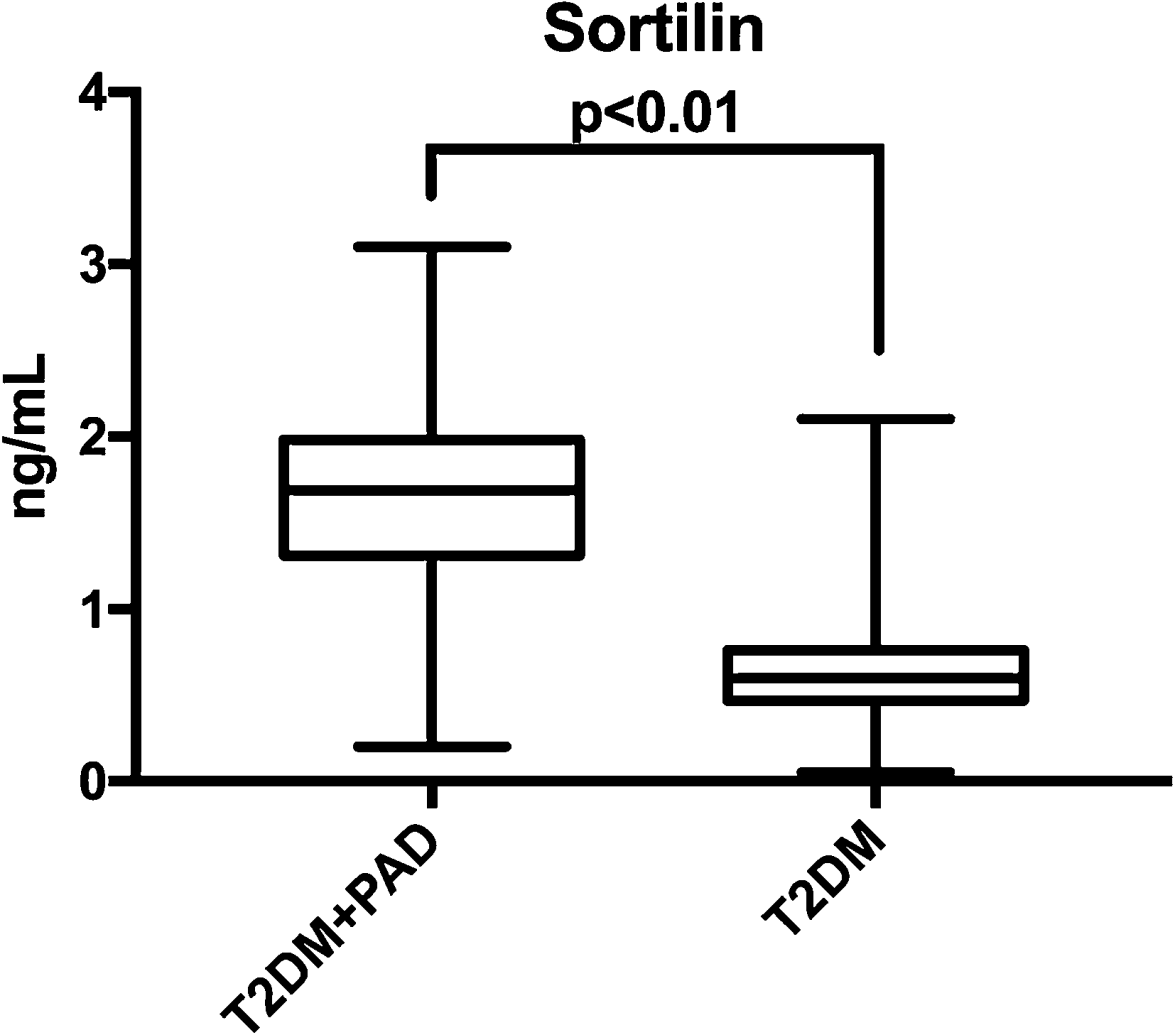
Sortilin levels according to PAD diagnosis. On the box plots, central lines represent the median, the length of the box represents the interquartile range and the lines extend to minimum and maximum values

**Fig. 2:**
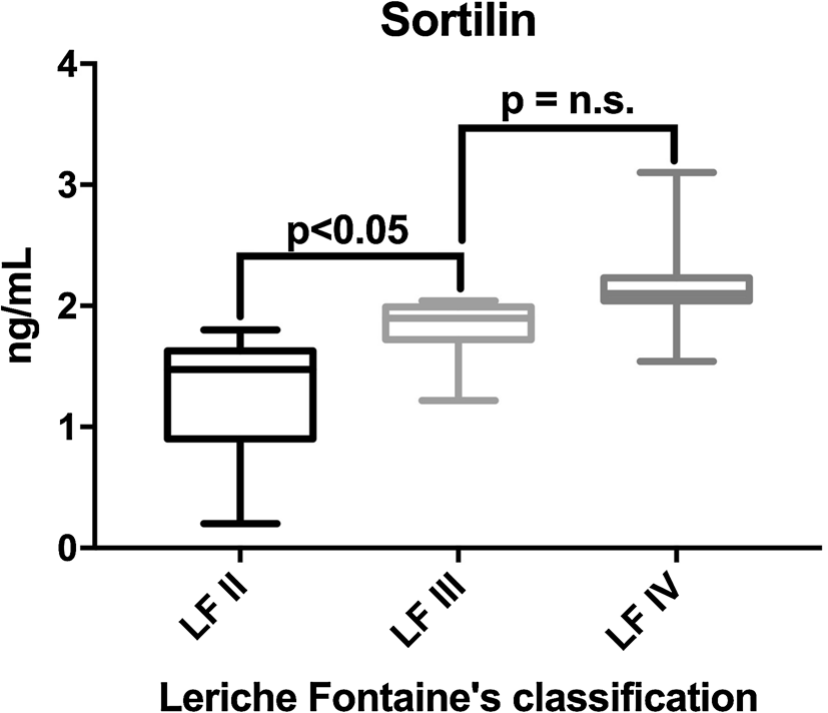
Sortilin levels according to PAD severity. On the box plots, central lines represent the median, the length of the box represents the interquartile range and the lines extend to minimum and maximum values

Sortilin concentration was higher among patients with PAD than among those without PAD (1.61 ± 0.54 ng/mL versus 0.67 ± 0.30 ng/mL, P < 0.01), as highlighted in Fig. 1. Moreover, when evaluating sortilin concentration according to patients’ functional status, a trend was evident, with higher levels of circulating sortilin in patients with more advanced disease (Fig. 2).

The multivariate logistic regression analysis showed that, after adjustments for cardiovascular risk factors, male gender, smoking, hypertension, hyperlipidemia, total cholesterol, HDL-cholesterol, LDL-cholesterol and sortilin levels were independent determinants for PAD occurrence in patients with T2DM (Table 4).

**Table 4:** Multivariable stepwise logistic regression model for presence of PAD for common risk factors and for sortilin

	Variable OR (95% CI)	P value
Age	1.13 (0.9–1.21)	0.187
Men	1.98 (1.12–2.78)	< 0.001
Smoking	33.51 (8.22–145.27)	< 0.001
Hypertension	19.97 (6.36–67.61)	< 0.001
Hyperlipidemia	18.42 (5.15–31.38)	0.003
Total cholesterol	1.13 (0.98–2.14)	0.012
HDL-cholesterol	0.09 (0.02–0.18)	0.211
LDL-cholesterol	11.33 (4.15–62.14)	0.055
Sortilin	5.21 (1.92–18.32)	0.003

**Fig. 3:**
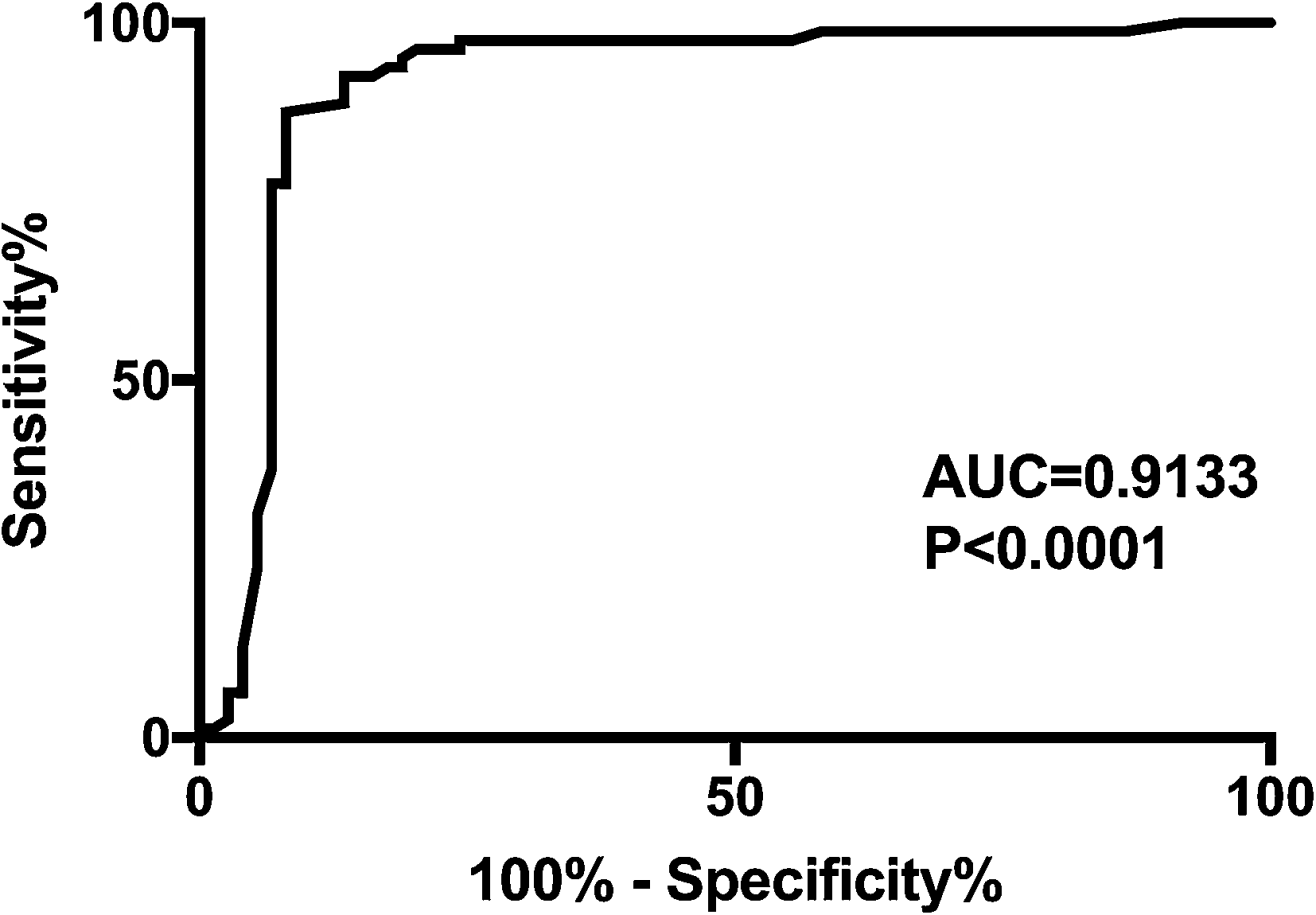
ROC curve analysis of the ability of sortilin to predict the presence of PAD in diabetic patients

The ability of the area under the ROC curve based on sortilin levels to predict the presence of PAD in diabetic patients was 0.9133 (Fig. 3).

## Discussion

The ABI is a very commonly used tool, easy to operate and it is able to identify PAD very effectively. When ABI is suggestive of PAD, however, we are often faced with a patient with an already advanced disease [39]. If we consider that PAD in diabetic patients can be even more aggressive and can manifest the symptoms later, the research for tools useful for an early diagnosis appears essential [40]. There are some potential biomarkers able to identify a subset of diabetic patients with PAD more prone to suffer major amputations; for instance, the tumor necrosis factor receptor 1 (TNFR1) has been associated with major amputation outcome [41]. Another possible biomarker for the presence of PAD in diabetic patients is represented by the high mobility group box 1 (HMGB1) which, together with the osteoprotegerin (OPG), has been associated with the presence of PAD in these patients [42]. Furthermore, also the fibroblast growth factor (FGF) 23 has been correlated with the presence of lower extremity atherosclerotic disease in diabetic patients [43]. In this scenario, sortilin may be of clinical interest as a simple, easy to measure biochemical marker for atherosclerotic disease. Numerous evidence suggests that sortilin is implicated in the pathogenesis of many inflammatory and metabolic diseases, including T2DM and atherosclerotic complications of diabetes [31]. In addition, sortilin has been shown to be an energetic regulator of lipid metabolism [44]. No definitive data about the role of sortilin in atherosclerosis and diabetes, however, are available. We have evidence that sortilin is decisive in the formation of atherosclerotic plaque and that therapeutic agents capable of reducing serum sortilin levels are able to improve atherosclerosis [30]. Sortilin-dependent uptake of LDL into macrophages represents an interesting mechanism of foam cell formation, the fundamental lesion of atherosclerotic disease [21]. There are data demonstrating that liver sortilin, however, could play a positive role in atherosclerosis, improving cholesterol metabolism and reducing LDL levels, which are fundamental players in the formation of atherosclerotic lesion [15]. Recent data have shown that a process dependent on autophagy degrades apoB that was diverted from the secretory pathway by sortilin and provides a mechanism contributing to the reduction of LDL cholesterol [45]. It is therefore possible that sortilin performs different functions depending on the district where it is produced, on the receptors which it binds and on the extracellular milieu where it is released. Oh and colleagues [26] have shown that this protein is associated with coronary artery disease and diabetes. To our knowledge, this is the first time that the sortilin serum concentration has been assessed as a potential biomarker for peripheral atherosclerotic artery disease of the lower limbs in a diabetic population. Our study shows a strong correlation between sortilin concentration and lower limb clinically significant atherosclerotic disease in T2DM patients. Previous experimental evidence has shown that sortilin is involved in the formation of atherosclerotic plaque. In particular, in a mouse model it has been shown that sortilin facilitates plaque formation by promoting the inflammatory setting [22]. It is therefore possible that in our population of diabetic patients, high sortilin values are responsible, at least in part, for atherosclerotic lesions of the lower limbs. An additional important data is that the levels of sortilin correlate with the PAD severity in diabetic patients, as if there were a dose-dependent relationship. Of particular interest, there is very little overlap between sortilin concentration in patients with PAD and in patients without PAD. The ROC curve also confirms that sortilin levels are able to predict the presence of PAD in our population of diabetic patients. If such result was confirmed, sortilin concentration could prove a biomarker with excellent sensitivity and specificity for peripheral artery disease in diabetic subjects.

A strength of this study is the fact that we enrolled only patients who were not on statin therapy. Given that statin therapy may affect sortilin concentration, our result, thus, are unlikely to be due to interference of pharmacological therapy. On the other hand, as many patients with diabetes or at high cardiovascular risk are more likely to be on statin therapy, our results need to be confirmed in a larger, more representative population. Statin therapy has been shown to reduce statin levels in patients with CAD [30]. Even circulating PCSK9 has been independently related to sortilin, and also their correlation is affected by the statin therapy [46]. Since our patient population was statin-free, the data we have documented are not affected by this aspect. At the same time, this finding could reinforce the hypothesis of using this biomarker to stratify patients, both from a diagnostic and a therapeutic point of view. It would be justified to assess PAD in diabetic patients with higher sortilin levels and it would be useful to start a more aggressive statin treatment in this subset of patients.

It is not possible to definitively clarify whether sortilin levels are a cause or effect of atherosclerosis, or even both, in a sort of vicious circle. The earlier studies on sortilin highlighted its causative role in atherosclerotic disease, mainly due to the action of intracellular sortilin. In fact, in the study by Musunuru and colleagues [15], protective SNP was associated with a reduced intracellular concentration of sortilin and it was this intracellular action that is thought as associated with atherosclerosis. It has been later shown, however, that sortilin is actively secreted by activated platelet and may play a role in foam-cell formation. Thus, circulating sortilin concentration may also be a consequence of atherosclerosis.

A limitation of our study is that its cross-sectional nature is not able to establish causal relationship between the findings. Even so, our result lend support to the hypothesis that sortilin actions in extra-hepatic tissue are important in the atherosclerosis physiopathology. A further limitation is the small number of patients included in the study, due to the particular condition that required the presence of diabetes and the absence of statin treatment. Another major limitation of our study is the lack of prospective data. We didn’t aim, however, to investigate sortilin as a maker for increased risk, we tested it as a marker for active disease. A further limitation of our study is in that we chose to define PAD as a clinically apparent disease. In fact, to overcome this limitation a prospective study would be needed. We believe that our present result may lay the foundation for such a subsequent prospective study.

## Conclusions

We demonstrated that circulating sortilin levels are associated with PAD in diabetic patients, that sortilin levels correlate with disease severity and that, therefore, sortilin is a promising marker for PAD in diabetic patients. Furthermore, in addition to risk factor-based validated scores, sortilin may help clinicians to better stratify atherosclerotic risk in diabetic patients. Further studies to confirm this hypothesis are needed and warranted.

## Abbreviations

ABI: ankle-brachial index
BMI: body mass index
CT: computed tomography
CAD: coronary artery disease
eGFR: estimated glomerular filtration rate
LDL: low-density lipoprotein
LL-PAD: lower limb PAD
MDRD: modification of diet in renal disease
PAD: peripheral artery disease
ROC: receiver-operating characteristics
R: receptor
TC: total cholesterol
T2DM: type 2 diabetes mellitus
VLDL: very low-density lipoprotein
